# The Specialist and the Practitioner

**Published:** 1897-04-10

**Authors:** 


### The Specialist and the Practitioner.

For years past the wail has gone up from the ranks of the general practitioners that the bread was being taken out of their mouths by the specialists. That the complaint was founded upon fact there can be no doubt, and that the loss to the general practitioner has been enormous is certain. But in the very multiplication of specialties comes the Nemesis of specialism.

The growth of specialism in these latter days has been so great that not only general practitioners, but physicians, and even surgeons, find themselves ousted from their work on every side by the intrusion of little specialties which a few years ago were almost unheard of. No longer does a patient expect to be sent to a neurologist, or to an ophthalmologist; to an operating surgeon or to a gynæcologist. Each of these, and each of the score other specialties which one might mention, is broken up into other still smaller departments, till nowadays general practitioners are almost as likely to get into hot water with their patients for sending them to the wrong specialist as formerly they were for not sending them to a specialist at all. It is, however, in this sub-division that we foresee the decadence of specialism, at anyrate in its more offensive forms.

The real scientific objection to specialism has always been that the increase of knowledge gained in regard to each portion of the body was obtained at the expense of a lessened grasp in regard to the whole, and those who have taken broad and philosophical views of medicine have been able to quote endless instances of the errors arising from "specialist" attempts to treat just that portion of the frame which happened to ache the most. This is a positive and insuperable objection to specialism in medicine, which never has and probably never will be got over, and it applies with infinitely greater force to that sub-division of specialties which is so marked a feature in modern days.

There are two influences which tend greatly to alter the standing of the specialist, and dethrone him from his high position; one the greatly improved education of the modern student in all the knowledge which used to be special to the specialist, the other, the narrowing of specialisms to such small and petty dimensions as to render those who practise them mere journeymen for the performance of special "jobs." Some time ago events seemed to be drifting in the direction of the specialist becoming master of the situation, and taking entire charge of his patients. The man who thought his main ailment lay in his liver employed the liver specialist as his ordinary medical adviser, and the general practitioner was relegated to attendance on trivial ailments. Now, however, the trend of affairs is different; the general practitioner is educated in the same knowledge, and uses the same instruments as the specialist, and does not appear to the same disadvantage in his patients' eyes as did his predecessor, whose knowledge was that of pre-specialist times, and whose instrumental accessories consisted of his wonderful "pocket case," and of the stethoscope which decorated the interior of his hat. On the other hand, instead of a patient being able to consult a single specialist suited to the whole of his case, he has indeed to suffer many things from very many physicians if he is to obtain all the advice which modern specialism can afford for the varying phases of his disorder. Herein lies the opportunity of the general practitioner, who, if he is wise, will use the knowledge with which he is provided by modern education to keep the specialist in his proper place. If the modern, well-educated practitioner plays his cards well, the specialist in future will take much the same position as is now occupied by the dentist—a most useful and praiseworthy person for his own work, to be employed when he is wanted, and then put on one side.

"And who is your doctor, my dear?" says the ancient aunt. "Well," says the modern young lady, "my real doctor is Dr. Jones, but when I have a baby he employs Dr. A——, and when my back was bad he got Dr. B—— to see me, and when I wanted some glasses he sent me to Dr. C——, and when I had an abscess he had some one in from Harley Street—I never heard his name; and then there was a nerve man from Cavendish Square when the baby had fits, and for my teeth I generally go to Dr. D——, but Dr. E—— pulled Johnny's tooth because Dr. Jones thought he did extractions better; then, you know, there is the man who cuts my corns, and, of course, there is the massage woman. I really do not know all these people. One's doctor can always find some one to do these sort of things, and Dr. Jones is very clever and always employs nice people." And such threatens to be the end of specialism—fallen by being sub-divided.

